# Incidence and outcome of cardiac injury in patients with severe head trauma

**DOI:** 10.1186/s13049-016-0246-z

**Published:** 2016-04-27

**Authors:** Ahmed Hasanin, Amr Kamal, Shereen Amin, Dina Zakaria, Riham El Sayed, Kareem Mahmoud, Ahmed Mukhtar

**Affiliations:** Department of Anesthesia and Critical Care Medicine, Faculty of Medicine, Cairo University, Cairo, Egypt; Department of Clinical and Chemical Pathology, Faculty of Medicine, Cairo University, Cairo, Egypt; Department of Cardiology, Faculty of Medicine, Cairo University, Cairo, Egypt

**Keywords:** Severe head injury, Cardiac injury, Echocardiography, Tsroponin

## Abstract

**Background:**

Although cardiac injury has been reported in patients with various neurological conditions, few data report cardiac injury in patients with traumatic brain injury (TBI). The aim of this work is to report the incidence of cardiac injury in patients with TBI and its impact on patient outcome.

**Methods:**

A prospective observational study was conducted on a cohort of 50 patients with severe TBI. Only patients with isolated severe TBI defined as Glascow coma scale (GCS) < 8 were included in the study. Acute physiology and chronic health evaluation (APACHE) II score, GCS, hemodynamic data, serum Troponin I, electrocardiogram (ECG), and echocardiographic examination, and patients’ outcome were recorded. A neurogenic cardiac injury score (NCIS) was calculated for all patients (rising troponin = 1, abnormal echocardiography = 1, hypotension = 1). Univariate and multivariate analyses for risk factors for mortality were done for all risk factors.

**Results and discussion:**

Fifty patients were included; age was 31 ± 12, APACHE II was 21 ± 5, and male patients were 45 (90 %). Troponin I was elevated in 27 (54 %) patients, abnormal echocardiography and hypotension were documented in 14 (28 %) and 16 (32 %) patients, respectively. The in-hospital mortality was 36 %. Risk factors for mortality by univariate analysis were age, GCS, APACHE II score, serum troponin level, NCIS, and hypotension. However, in multivariate analysis, the only two independent risk factors for mortality were APACHE II score (OR = 1.25, 95 % confidence interval: 1.02–1.54, *P* = 0.03) and NCIS score (OR = 8.38, 95 % confidence interval: 1.44–48.74, *P* = 0.018).

**Conclusions:**

Cardiac injury is common in patients with TBI and is associated with increased mortality. The association of high NCIS and poor outcome in these patients warrants a further larger study.

## Background

Neurogenic stress cardiomyopathy (a common form of stress-related cardiomyopathy) has been described in various neurological and neurosurgical conditions. Many authors have reported brain-heart interactions in subarachnoid hemorrhage (SAH) [1–8], ischemic or hemorrhagic cerebrovascular strokes [9–11], status epilepticus [12], central nervous system (CNS) infections [13], brain tumors [14], and various stressful events [15].

In cases of traumatic brain injury (TBI), neurocardiogenic injury was documented in few case reports [16] and case series [17] with no information about patient outcome. Only one study reported cardiac injury in TBI patients [18]; however, its retrospective design might preclude its ability to define the actual incidence rate and outcome of cardiac injury in this population.

This prospective observational study was designed to determine the incidence of cardiac injury and related mortality in patients with severe TBI.

## Methods

This prospective, observational study was conducted in the 24-bed trauma-surgical ICU at Cairo University Hospital. The study protocol was approved by the Research Ethics Committee, and informed consent was obtained from next-of-kin of patients prior to commencement.

Fifty patients with isolated severe TBI, defined as Glasgow coma scale (GCS) < 8, were consecutively included between January 2014 and February 2015.

Patients aged above 50 years and below 18 years, patients with a history of cardiac morbidities, those with chest, abdominal trauma, long bone fractures, history of cardiac arrest before or within 24 h of ICU admission, and those with hypovolemic, obstructive or septic hemodynamic instability were excluded from the study.

### Patient management and data collection

Upon admission to ICU, patients’ GCS scores were recorded. Temperature, non-invasive and invasive arterial blood pressure, five-lead electrocardiography, hourly urinary output and central venous pressure (CVP) were monitored in all patients. Day-to-day investigations had been carried out to monitor and individualize treatment. Endotracheal intubation and mechanical ventilation were indicated to protect airway and maintain PaCO2 between 35 and 40 mmHg. Medical (and/or) surgical treatment was performed, supervised by the attending intensivist and neurosurgeon.

Collected demographic and clinical information included age, gender, severity scoring system via acute physiology and chronic health evaluation II (APACHE II), APACHE IV, and the type and site of lesion. Hemodynamic data that included heart rate, respiratory rate, temperature, and arterial blood pressure were continuously monitored. GCS was recorded every day until patient death or discharge from ICU. For patients who needed sedation, GCS was assessed 3-4 h after stoppage of sedation. Sedation was done by propofol and fentanyl.

Cardiac assessment for the patients included a 12-lead ECG every 24 h, and serum troponin I on days 1 and 3. Trans-thoracic echocardiography was done within 12 h of admission, and on days 3, 5, and 7.

### Echocardiographic examination

A phased array probe with a frequency of 4 MHZ (Mindray diagnostic ultrasound system, model DC-N6) was used for echocardiographic examination. Assessment was done by two physicians with at least one-year experience in echocardiographic examination. Assessments of global LV contractility and regional wall motion abnormality (RWMA) were obtained by “eyeballing” from the parasternal long- and short-axes, apical two, apical three, apical four-chamber and subcostal views. Ejection fraction (EF) and fractional shortening (FS) were measured by applying M-Mode on the left ventricle at the tip of mitral leaflets or on the left ventricular mid-papillary level (Teichholz method) from both parasternal long- and short-axes views or subcostal views [19, 20].

### Troponin measurement

Cardiac Troponin I (cTnI) was assayed using SIEMENS Dimension EXL 200 integrated chemistry system based on LOCI technology. The reference interval for Troponin I was 0.000-0.056 ng/mL.

### Primary outcome

Primary outcome was the incidence of cardiac injury and its association with patient outcome. Myocardial injury was mainly diagnosed by trans-thoracic echocardiographic (TTE) examination of the left ventricular systolic function on day 1 (systolic dysfunction was defined as diminished left ventricular ejection fraction (EF) of less than 55 %).

### Secondary outcomes

- Hemodynamic data: Heart rate and systolic blood pressure daily. Tachycardia was considered if heart rate is above 100 bpm. Bradycardia was considered if heart rate is below 60 bpm. Hypertension was defined by “systolic BP above 140 mmHg or diastolic BP was above 90 mmHg”. Hypotension was defined by “systolic BP less than 90 mmHg, diastolic BP less than 50 mmHg or mean BP less than 65 mmHg”.
- Myocardial injury:
  - Serum Troponin I.
  - Any ECG abnormality.
- Seven- and thirty-day survival.
- In-hospital mortality.

We introduced neurogenic cardiac injury score (NCIS) calculated via three components: the presence of cardiac dysfunction (diagnosed by TTE), elevated serum troponin, and the occurrence of hypotension (without evidence of hypovolemia or sepsis). Assessment of the presence of the three components of NCIS was done during the first 3 days of ICU admission. One point was added for a “yes” answer to each of the aforementioned items. The minimum NCIS was zero and the maximum NCIS was 3. The relationship between NCIS and patient outcome (in-hospital mortality) was analyzed.

### Statistical analysis

Statistical analysis was performed using SPSS 15 (Chicago, IL). Categorical data were presented as frequency (%); continuous data were checked for normal distribution by inspection of histogram distribution. Normally distributed continuous data were presented as mean ± SD, and abnormally distributed data were presented as median (interquartile range). Patients were classified into survivors and non-survivors; Chi-square test was used to compare frequencies between the two groups. Unpaired *t*-test and Mann–Whitney test were used to compare the means for continuous data as appropriate. Multivariable logistic regression was used. Variables with a p value of less than 0.2 in univariate analysis were included in multivariate analysis. Variables with a *p* value of < 0.05 in multivariate analysis were considered statistically significant.

## Results

**Fig. 1:**
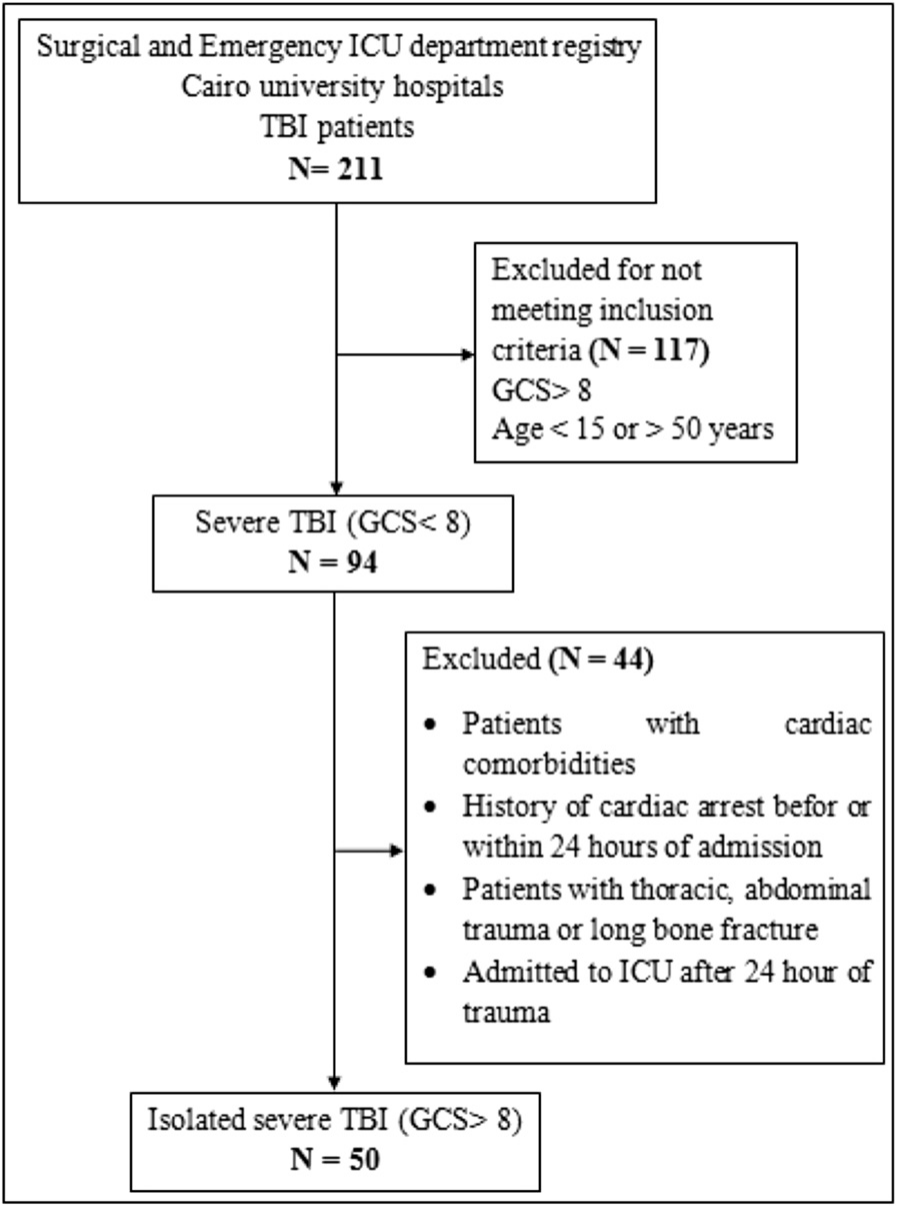
Flowchart of patient sampling. ICU: intensive care unit, TBI: traumatic brain injury, GCS: Glasgow coma scale

Two hundred eleven patients were admitted to our ICU with TBI in a period of 14 months. Ninety-four patients of severe head trauma (GCS < 8) had met our criteria and were enrolled in the study. Thirty patients were excluded due to presence of other injuries; 11 patients were excluded due to being above 50 years old; and three patients were excluded due to a history of cardiac arrest before ICU admission. Fifty patients were included in the final analysis, with 45 (90 %) of them being males, with a mean age of 30.8 ± 12.1 (Fig. 1).

The primary traumatic brain lesions were as follows: intracranial hemorrhage in 38 (72 %) patients, brain contusions in 8 (16 %) patients, and diffuse axonal injury in 4 (8 %) patients. Types of intracranial hemorrhage were extradural hemorrhage in 12 patients (24 %), subdural hemorrhage in 10 patients (20 %), subarachnoid hemorrhage in five patients (10 %), intracerebral hemorrhage in four patients (8 %), intraventricular hemorrhage in two patients (4 %), and mixed pathology in five patients (10 %). The mean GCS on admission was 6 with interquartile range [4–7]. Twenty-three patients (46 %) underwent surgical intervention. The mean score of the Acute physiology and chronic health evaluation II (APACHE II) was 21.1 ± 5.4 and that of the acute physiology and chronic health evaluation IV (APACHE IV) was 77.1 ± 22.5. Among the 50 patients, 13 (26 %) patients died within 7 days. However, the total in-hospital mortality was 18 (36 %) patients (Table 1).

**Table 1:** Demographic data; Data are presented as mean ± SD, median (IQR), and frequency (%)

Clinical characteristic	Value
Age	30.8 ± 12.1
Male gender	45 (90 %)
GCS	6 (4, 7)
APACHE II	21.1 ± 5.4
APACHE IV	77.1 ± 22.5
Main pathology	
• Hemorrhage	38 (76 %)
• Brain contusions	8 (16 %)
• Diffuse axonal injury	4 (8 %)
Surgical intervention	23 (46 %)
Seven day mortality	13 (26 %)
In-hospital mortality	18 (36 %)

*GCS*: Glasgow coma scale, *APACHE II*: Acute Physiology and Chronic Health Evaluation II score, *APACHE IV*: Acute Physiology and Chronic Health Evaluation IV score

The cardiac assessment of patients revealed that among the 50 patients, the ECG for 31 (62 %) patients showed abnormalities, [six (12 %) patients had abnormal QT interval, 29 (58 %) patients developed tachyarrhythmia, and 5 (10 %) patients developed sinus bradycardia] (Table 2). The measurements of troponin I enzyme level by day 1 were elevated in 27 (54 %) patients. The echocardiographic assessment for contractility revealed abnormalities in 14 (28 %) patients, five (10 %) of them were global hypokinesia. Sixteen (32 %) patients developed systemic arterial hypotension. The cardiac injury score was 0 in 16 (32 %) patients, 1 in 15 (30 %) patients, 2 in 14 (28 %) patients, and 3 in 5 (10 %) patients.

**Table 2:** Cardiac assessment: data are presented as mean ± SD, median (IQR), and frequency (%)

Clinical characteristic	Value
Elevated Troponin I by day 1	27 (54 %)
Any ECG abnormality	31 (62 %)
Abnormal Echocardiography	
• Any wall motion abnormalities	15 (30 %)
• Global hypokinesia	6 (12 %)
Hypotension	16 (32 %)
CIS	1 (0,2)
CIS	
• 0	16 (32 %)
• 1	15 (30 %)
• 2	14 (28 %)
• 3	5 (10 %)

*CIS*: cardiac injury score

Univariate analysis indicated that in-hospital mortality was associated with higher age (33.4 ± 12.7 versus 26 ± 9.6 years, *P* **=** 0.05), higher APACHE II score (23.6 ± 4.4 versus 16.8 ± 4.2, *P* < 0.001), lower GCS (6.4 ± 0.7 versus 5 ± 1.39, *P* < 0.001), higher NCIS score (1.6 ± 0.9 versus 0.38 ± 0.61, *p* < 0.001), higher serum Troponin level at day 1 (1.1 ± 2.3 versus 0.79 ± 1.7 ng/ml, *P* < 0.001) when compared to patients who survived. In-hospital mortality was also associated with brain hemorrhage (87.5 % versus 61.1 %, *P* = 0.041) and higher incidence of hypotension (46.9 % versus 5.6 %, *P* = 0.004) compared to patients who survived (Table 3).

**Table 3:** Risk factors for in-hospital mortality: univariate analysis. Data are presented as mean ± SD, median (IQR), and frequency (%)

	Non-survivors (*n* = 18)	Survivors (*n* = 32)	*P* value
Age	33.4 ± 12.7	26 ± 9.6	0.05
Male gender	29 (90.6 %)	16 (88.9 %)	1
APACHE II score	23.6 ± 4.4	16.8 ± 4.2	<0.001
GCS score	4.5 (4, 6)	7 (6, 7)	<0.001
Abnormal Echocardiography	13 (40.6 %)	2 (11.1 %)	0.052
Abnormal ECG	24 (75 %)	11 (61.1 %)	0.073
Hypotension	15 (46.9 %)	1 (5.6 %)	0.004
Troponin Day 1	1.1 ± 2.3	0.09 ± 0.18	<0.001
Elevated Troponin by Day 1	22 (68.8 %)	5 (27.8 %)	0.008
CIS	2 (1, 2)	0 (0,1)	<0.001
CIS > 1	18 (56.3 %)	1 (5.6 %)	0.001

*GCS*: Glasgow coma scale, *CIS*: cardiac injury score

Multivariate logistic regression model showed that independent factors that affected mortality were GCS (OR = 0.18, 95 % CI: 0.041–0.869, *p* = 0.032), APACHE II score (OR = 1.26, 95 % CI: 1.028–1.550, *p* = 0.026), and NCIS (OR = 16.02, 95 % CI: 2.029–126.607, *p* = 0.009) (Table 4). Mortality rate was 94.7 % among patients with NCIS above 1 versus 45.2 % in patients with NCIS less than 1 {*P* = 0.001, OR = 21.8 (2.58–184.71)}.

**Table 4:** Risk factors for in-hospital mortality: multivariate analysis

Risk factor	Odds ratio	*P* value	95 % confidence interval	
			Lower limit	Upper limit
GCS	0.182	0.032	0.041	0.869
APACHE II	1.263	0.026	1.028	1.550
CIS	16.028	0.009	2.029	126.607

*GCS*: Glasgow coma scale, *CIS*: cardiac injury score

## Discussion

This study documented two main findings: First, cardiac injury occurs in nearly 50 % of patients with severe TBI. Second, there was an association between cardiac injury and poor outcome in patients with TBI; this association was linked to the severity of cardiac injury as assessed using NCIS.

Although brain-heart interactions were previously reported in many CNS conditions, only few studies highlighted the link between cardiac injury and TBI [16, 17]. Most of these aforementioned studies enrolled a small series of single cohort of patients with no data about the patients’ final outcome.

In the current study, half of the patients developed cardiac injury as documented by elevated Troponin I in 54 % of patients, abnormal ECG in 62 % of patients, and echocardiographic examination in 42 % of patients. The incidence of cardiac dysfunction reported by Prathep and colleagues [18] in patients with TBI is not in line with our results. They reported abnormal echocardiographic examination in 22.3 % of patients and elevated serum troponin in 24 % of patients, with no data about patients’ ECG abnormalities. This difference might be due to many factors. First, all patients included in the present study had severe TBI. In contrast, only 56 % in Prathep’s study [18] were diagnosed with severe TBI. Second, our patients had a mean age of 30.8 ± 12 years which is generally lower than the patients included in Prathep’s study [18] (58 ± 20 years). Finally, the prospective design of our study allowed serial echocardiographic examination three times and serum troponin in all patients. In contrast, because of the retrospective nature of Prathep’s study [18], only one echocardiographic examination was done in 95 % of their patients, and serum Troponin I was measured in only 22 % of their patients. Moreover, these measurements were driven by clinical needs and not by study design that might underestimate the actual incidence of cardiac injury.

Serri et al. [21] recently reported a low incidence of cardiac injury among 41 patients with severe TBI. Serri’s findings differed from our reports as well as Prethep’s findings, this difference warrants more studies with larger sample size to investigate the actual incidence of cardiac injury in severe TBI.

Many theories have been proposed to explain stress-induced cardiac injury in acute CNS conditions; the most widely accepted one is catecholamine-mediated direct cardiac injury “catecholamine hypothesis” as a result of autonomic stimulation caused by direct brain injury. Many authors have reported elevated levels of catecholamines in the serum of patients with SAH [8, 22], in an experimental model of SAH in dogs [23], and also in cases of myocardial stunning after sudden emotional stress [15].

In the current study, we reported an increased incidence of in-hospital mortality in TBI patients with cardiac injury. Consistent with our findings, cardiac injury was a mortality risk factor in patients with TBI [18], CVS [9–11], and SAH [24–26]. Some authors reported cardiac injury to be a reversible condition that improves over time [7].

In our study, we introduced a novel scoring system (NCIS) for grading the severity of cardiac injury in patients with TBI. Formulation of the components of NCIS was based on three questions: 1- Is there an evidence cardiac injury? The elevation of Troponin I fulfils this question. 2- Does this cardiac injury affect the cardiac function? The presence of echocardiographic findings fulfils this question. 3- Does this cardiac injury affect the patient’s hemodynamic status? The presence of hypotension fulfils this question.

We reported an association between higher NCIS and poor outcome. Mortality was 94.7 % among patients with NCIS above 1 versus 45.2 % in patients with NCIS less than 1 (*P* = 0.001). This finding might raise the attention toward further research in larger studies to validate this score and applying it in other populations with neurological conditions.

Our findings have several potential clinical implications in both intensive care and anesthetic practice in cases of severe TBI. The awareness of the possible presence of cardiac injury in these patients and its impact on patient outcome might raise the attention toward cardiac protection in cases with higher risk. Although there is no consensus till now about the efficacy of any mode of cardiac protection in neurosurgical patients [27], a possible role for propranolol and phentolamine in cardiac protection was suggested by Neil-Dwyer and colleagues [28] who reported the absence of myocardial necrosis in post-mortem specimens for patients with SAH who received these drugs. However, this finding had no impact on patient outcome. The presence of cardiac injury most likely reflects a severe TBI and consequently predicts a poor outcome. It is therefore less likely that the efforts to treat the cardiac condition will influence the outcome.

Our results might also help in risk stratification and in taking precautions in anesthetic management of these cases to avoid further deterioration in the patient hemodynamics. The unexpected high troponin level in this population may warrant changing the standard perioperative cardiac assessment of these patients before surgery. However, this hypothesis needs more validation in larger studies.

Our study had some limitations. The first limitation is the relatively small sample size; however, most of the previous studies reporting cardiac injury included a number near to ours. We excluded patients above 50 years old to avoid enrollment of patients with undiagnosed ischemic heart disease. We also excluded patients with any other injuries to avoid involving any patients with chest trauma affecting cardiac function and patients with any other type of shock that might affect data analysis. Exclusion of some subgroups of patients (elderly patients and multiple trauma patients) might affect the generalizability of our results, and make our findings restricted to patients with isolated head trauma. We did not report any injury severity score for our patients. This was potentially compensated by using ICU severity scores (APACHE II and APACHE IV). We did not use cardiac markers other than Troponin such as creatine kinase and brain natriuretic peptide.

## Conclusions

Our study demonstrated that cardiac injury is a common event in patients with TBI and is associated with increased mortality. The association of high NCIS and poor outcome in these patients warrants further investigation.

### Key messages

- Cardiac injury is common in patients with severe head trauma; it is associated with increased mortality.
- Neurogenic cardiac injury score is a new score introduced to categorize the severity of cardiac injury in severe head trauma; it is a simple score based on serum troponin elevation, abnormal echocardiographic findings, and hypotension.
- There is an association between high neurogenic cardiac injury score and poor outcome (especially when the NCIS is above 1); this association warrants further investigation.

## Abbreviations

APACHE: Acute physiology and chronic health
BP: blood pressure
CNS: central nervous system
cTnI: troponin I
CVP: central venous pressure
ECG: electrocardiogram
F: Ejection fraction
FS: Fraction shortening
GCS: Glasgow coma scale
HR: heart rate
ICU: Intensive care unit
MHZ: megahertz
NCIS: neurogenic cardiac injury score
OR: Odds ratio
PaCo_2_: partial pressure of CO_2_
ROC: receiver operating characteristic
RWMA: regional wall motion abnormalities
SAH: subarachnoid haemorrhage
TBI: traumatic brain injury
TTE: trans-thoracic echocardiography
