# Erratum to: Incidence and outcome of cardiac injury in patients with severe head trauma

**DOI:** 10.1186/s13049-016-0263-y

**Published:** 2016-05-25

**Authors:** Ahmed Hasanin, Amr Kamal, Shereen Amin, Dina Zakaria, Riham El Sayed, Kareem Mahmoud, Ahmed Mukhtar

**Affiliations:** Department of Anesthesia and Critical Care Medicine, Faculty of Medicine, Cairo University, Cairo, Egypt; Department of Clinical and Chemical Pathology, Faculty of Medicine, Cairo University, Cairo, Egypt; Department of Cardiology, Faculty of Medicine, Cairo University, Cairo, Egypt

## Erratum

After publication of the original article [1], the authors noticed two errors affecting one of the Keywords, and Tables 2–4.

A minor typo in the keyword ‘Troponin’ was unfortunately introduced while the manuscript was in production. The typo has been corrected in the original article.

Secondly, Tables 2, 3 and 4 contain an incorrect abbreviation. Prior to editorial acceptance of the article, ‘CIS’ (cardiac injury score) was revised to ‘NCIS’ (neurogenic cardiac injury score) throughout the manuscript, but this was missed for these Tables. In the original article, this abbreviation has been corrected to ‘NCIS’ in the body and footnote of each Table.
